# Genetically Encoded Biosensor-Based Screening for Directed Bacteriophage T4 Lysozyme Evolution

**DOI:** 10.3390/ijms21228668

**Published:** 2020-11-17

**Authors:** Seung-Gyun Woo, Seong Keun Kim, Baek-Rock Oh, Seung-Goo Lee, Dae-Hee Lee

**Affiliations:** 1 Synthetic Biology and Bioengineering Research Center, Korea Research Institute of Bioscience and Biotechnology (KRIBB), Daejeon 34141, Korea; dntmdrbs12@kribb.re.kr (S.-G.W.); draman97@kribb.re.kr (S.K.K.); 2 Department of Biosystems and Bioengineering, KRIBB School of Biotechnology, University of Science and Technology (UST), Daejeon 34113, Korea; 3 Microbial Biotechnology Research Center, Jeonbuk Branch Institute, Korea Research Institute of Bioscience and Biotechnology (KRIBB), Jeongeup 56212, Korea; baekrock.oh@kribb.re.kr

**Keywords:** bacteriophage T4 lysozyme, genetically encoded biosensor, directed evolution

## Abstract

Lysozyme is widely used as a model protein in studies of structure–function relationships. Recently, lysozyme has gained attention for use in accelerating the degradation of secondary sludge, which mainly consists of bacteria. However, a high-throughput screening system for lysozyme engineering has not been reported. Here, we present a lysozyme screening system using a genetically encoded biosensor. We first cloned bacteriophage T4 lysozyme (T4L) into a plasmid under control of the *araBAD* promoter. The plasmid was expressed in *Escherichia coli* with no toxic effects on growth. Next, we observed that increased soluble T4L expression decreased the fluorescence produced by the genetic enzyme screening system. To investigate T4L evolution based on this finding, we generated a T4L random mutation library, which was screened using the genetic enzyme screening system. Finally, we identified two T4L variants showing 1.4-fold enhanced lytic activity compared to native T4L. To our knowledge, this is the first report describing the use of a genetically encoded biosensor to investigate bacteriophage T4L evolution. Our approach can be used to investigate the evolution of other lysozymes, which will expand the applications of lysozyme.

## 1. Introduction

Lysozyme has been widely used as a model protein to evaluate protein structure–function relationships [1], such as in studies of amyloid fibril disease [2] and as a preservative for foods and over-the-counter medicines [3]. Recently, lysozyme was used for biological treatment to improve the degradation of secondary sludge. Because secondary sludge mainly consists of concentrated microbial cells, conventional anaerobic digestion processes cannot degrade over 35% of organic substances in secondary sludge [4,5,6,7]. Therefore, biological hydrolysis of bacterial cells is a rate-limiting step in most anaerobic digestion processes for secondary sludge [8,9]. In conventional anaerobic digestion processes, multiple physicochemical approaches are used to enhance the hydrolysis efficiency of secondary sludge [10,11,12], including thermal pretreatment [13], ultrasonic technology [10], and acid/alkaline chemical pretreatment [14]. These physicochemical methods require large amounts of energy and corrosive equipment.

Enzymes have been shown to be effective for the biological pretreatment of secondary sludge [15]. Although proteases and amylases are widely used in secondary sludge pretreatment to improve biodegradation, enzymes that hydrolyze the cell walls of bacteria in secondary sludge have been identified as effective biocatalysts. Indeed, sludge digestion by lysozyme reduces excess sludge generation by almost 100% in serial batch reactors [16].

Lysozyme is a glycoside hydrolase that digests β-1,4-glycosidic bonds between β-1,4-linked *N*-acetyl-D-glucosamine and *N*-acetylmuramic acid of peptidoglycan (PG) in the bacterial cell wall [17]. Bacteriophage PG hydrolases, including glycosidases, *N*-acetylmuramoyl-L-alanine amidases (or amidase), and peptidases (or endopeptidases), catalyze disruption of the bacterial cell wall [18]. Each enzyme targets and cleaves a different covalent bond within the PG layer based on the catalytic active site [18]. β-1,4-glycosidic bonds within the PG layer are highly conserved in almost all bacterial species, whereas the covalent bonds of the PG layer that are hydrolyzed by amidases or endopeptidases differ between bacterial genera and species [19,20,21].

One reason for the wide use of lysozymes is their accessibility. Hen egg white lysozyme is mainly purified from eggs, and recombinant human lysozyme is produced in engineered plants. However, these methods cannot produce sufficient amounts of lysozymes for various applications. Moreover, the use of wild-type (WT) lysozymes is limited by their low activity during secondary sludge biodegradation. Many studies have been conducted to produce lysozymes in microbial hosts. For instance, *Saccharomyces cerevisiae* [22], *Kluyveromyces lactis* [23], and *Komagataella phaffii* (formerly known as *Pichia pastoris*) [24] have been used to produce recombinant human lysozyme. Although *Escherichia coli* is widely used as a cost-effective expression bacterial host to produce heterologous proteins [25], engineered *E. coli* strains expressing soluble lysozymes show rapid cellular lysis and low production yield [26]. Most previous studies have shown that lysozymes form inactive inclusion bodies in *E. coli* [27]; inefficient refolding and purification steps are required for these proteins to function.

Genetically encoded biosensors are key devices in synthetic biology and are used for high-throughput screening (HTS) of enzyme libraries [28,29]. We developed a genetic enzyme screening system (GESS) that uses a transcription factor-based genetic circuit with wide applicability in screening diverse enzymes from metagenomic or enzyme mutant libraries [28]. A GESS can detect the catalytic activity of various enzymes, including tyrosine phenol-lyase, lipase, cellulase, and methyl parathion hydrolase [28,29,30,31,32,33,34,35]. Furthermore, a GESS can be more generally applied because the designed substrates sense specific enzyme activity, whereas other genetic circuits use metabolite- or product-induced transcription systems to screen for specific enzymes.

In this study, we produced soluble and functional lysozyme from bacteriophage T4 (T4L) in *E. coli* and improved T4L activity via directed evolution using a GESS. We first constructed a T4L expression system that is tightly regulated by L-arabinose and examined the effects of soluble T4L expression on *E. coli* growth. Next, we generated a T4L mutant library by random mutagenesis and carried out GESS-based HTS of the library. Finally, we identified two T4L variants with improved activity.

## 2. Results and Discussion

### 2.1. Design of T4L Screening Using Genetic Circuits

**Figure 1:**
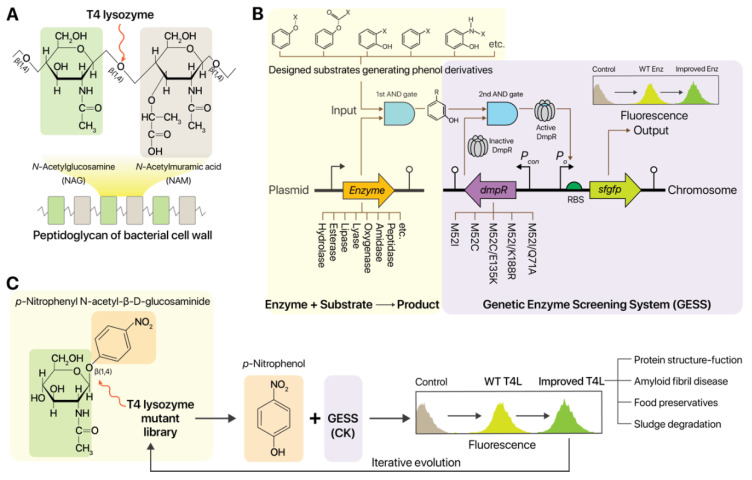
Design of the T4 lysozyme (T4L) screening system based on a genetic enzyme screening system (GESS). (**A**) T4L hydrolyzes the β-1,4-glycosidic bond between *N*-acetyl-D-glucosamine and *N*-acetylmuramic acid in bacterial cell wall peptidoglycans (PGs). (**B**) GESS for high-throughput enzyme screening. GESS detects the phenolic product generated by enzymatic catalysis of designed substrates. Enzymes include hydrolase, esterase, lipase, lyase, oxygenase, amidase, and peptidase. Enzyme activity is visualized using super-folder green fluorescence protein (sfGFP), whose expression is activated by DmpR binding to phenolic ligands. (**C**) GESS-based T4L screening. *p*-Nitrophenyl *N*-acetyl-β-D-glucosaminide was chosen as the designed T4L substrate and is composed of *p*-nitrophenol and *N*-acetyl-β-D-glucosaminide linked together by a β-1,4-glycosidic bond. When T4L catalyzes β-1,4-glycosidic bond hydrolysis in *p*-nitrophenyl *N*-acetyl-β-D-glucosaminide, *p*-nitrophenol is generated and detected by the DmpR-CK variant (M52C/E135K).

T4L breaks down the β-1,4-glycosidic bond between *N*-acetyl-D-glucosamine and *N*-acetylmuramic acid in the PG layer of the bacterial cell wall (Figure 1A). Although T4L is a model protein used to study protein structure–function relationships, an HTS system for T4L engineering has not been reported. Gram-positive bacterial cells are widely used to assay T4L activity, making it difficult to develop an HTS system for directed T4L evolution. A previous study screened combinatorial libraries of T4L in auxotrophic *E. coli* strains to investigate whether directed evolution of T4L generates T4L mutants with new enzyme activities such as β-galactosidase or prephenate dehydratase [36]. Previously, we reported a GESS-based HTS of enzymes and microbes from metagenomic and mutation libraries [28]. The transcription regulator DmpR used in the GESS utilizes various phenolic compounds as ligands for transcriptional regulation of a reporter gene, super-folder green fluorescence protein (*sfgfp*) (Figure 1B). These phenolic compounds can be released via catalysis of various substrates by multiple enzymes (hydrolase, esterase, lipase, lyase, oxygenase, amidase, and peptidase) (Figure 1B). To expand the ligand specificity of WT DmpR, a mutant library of WT DmpR was recently generated and screened, yielding five DmpR variants: M52I, M52C, M52C/E135K (CK), M52I/K188R (IR), and M52I/Q71A (IA) [37]. These DmpR variants showed lower background signals and wider dynamic ranges compared to WT DmpR [37]. To engineer T4L using these genetic circuits, we screened several synthetic compounds useful as T4L substrates. Among them, we adopted a synthetic substrate, *p*-nitrophenyl *N*-acetyl-β-D-glucosaminide, which is composed of *p*-nitrophenol (*p*NP) and *N*-acetyl-β-D-glucosaminide linked by a β-1,4-glycosidic bond. When T4L catalyzes the hydrolysis of the β-1,4-glycosidic bond in *p*-nitrophenyl *N*-acetyl-β-D-glucosaminide, *p*NP is generated and detected by the DmpR-CK variant (Figure 1C). A single copy of the GESS-CK construct was chromosomally integrated into *E. coli* DH5α cells to minimize background noise, resulting in an *E. coli* DH5α-GESS-CK strain [37].

### 2.2. Tunable T4 Lysozyme Expression

**Figure 2:**
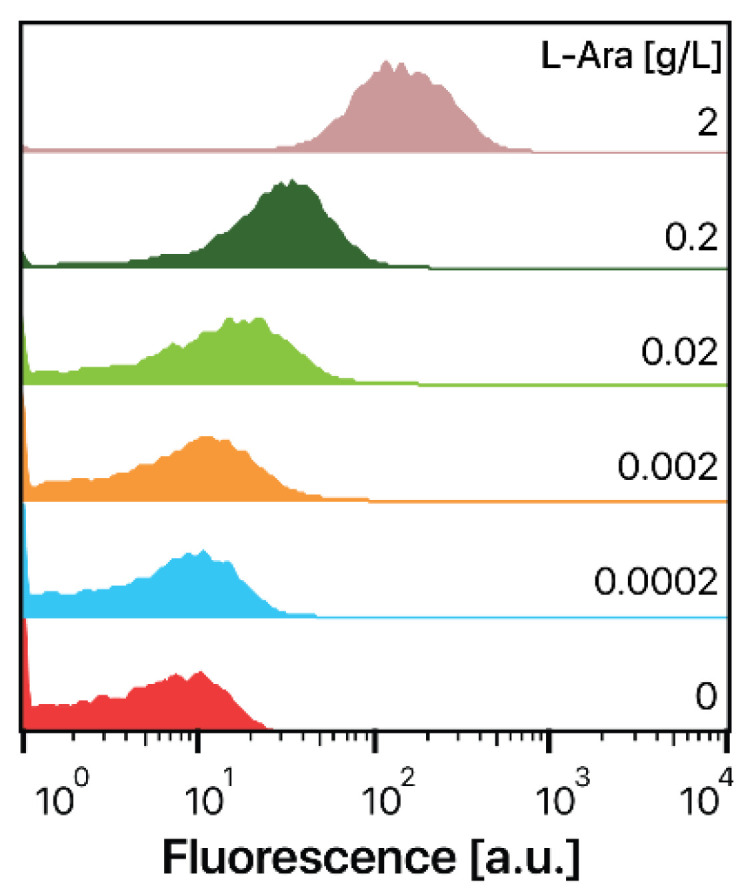
Tunable expression of sfGFP in *E. coli* DH5α. *E. coli* DH5α cells harboring the pBAD-sfGFP plasmid were grown in the presence of different concentrations of L-arabinose (0–2 g/L) at 37 °C for 8 h, and fluorescence was determined by flow cytometry.

To screen the T4L mutant library using the GESS and designed substrate, we first examined T4L expression in *E. coli*, as T4L expression often leads to *E. coli* host cell lysis [19]. To express T4L in the *E. coli* DH5α strain, we adopted a tightly regulated *araBAD* promoter (P*_BAD_*) to avoid leaky expression in *E. coli* [38]. To determine whether the P*_BAD_* promoter could be used for tunable T4L expression in the *E. coli* DH5α strain, DH5α cells harboring pBAD-sfGFP were induced by 0–2 g/L L-arabinose. Heterologous genes regulated by the P*_BAD_* promoter are expressed in an all-or-none pattern because of the *E. coli* L-arabinose transporter, resulting in a bimodal distribution of *E. coli* populations [39,40]. However, we detected dose-dependent sfGFP expression after L-arabinose treatment, and sfGFP was uniformly expressed at each dose of L-arabinose (Figure 2), indicating that the P*_BAD_* promoter can be used for tunable T4L expression in *E. coli* DH5α cells. 

**Figure 3:**
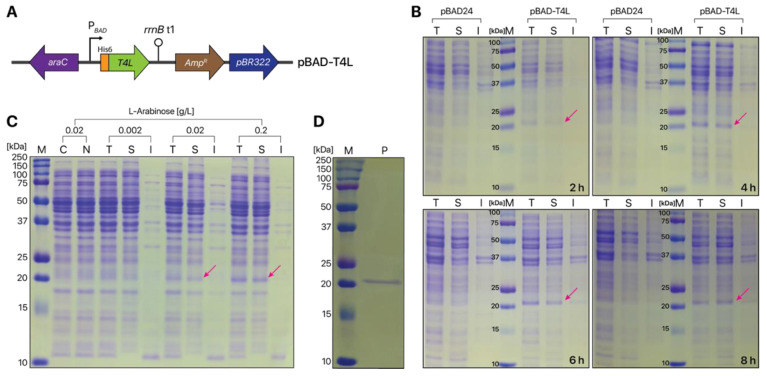
T4L expression in *E.* *coli*. (**A**) pBAD-T4L plasmid map. The plasmid contains the T4L gene, which is expressed under control of the P*_BAD_* promoter. (**B**) Time course monitoring of T4L expression. DH5α cells harboring the pBAD-T4L or pBAD24 (control) plasmid were grown in the presence of 0.02 g/L L-arabinose to induce T4L expression, which was monitored for 8 h. (**C**) SDS-PAGE analysis of T4L expression in the presence of different L-arabinose concentrations. Recombinant T4L was expressed by DH5α cells in the presence of 0.002, 0.02, and 0.2 g/L L-arabinose, followed by 15% SDS-PAGE analysis. Two negative controls (C: *E. coli* DH5α cells, N: *E. coli* DH5α cells containing the pBAD24 empty plasmid) were analyzed in the presence of L-arabinose (0.02 g/L) to explore leaky T4L expression. (**D**) Purification of T4L expressed in DH5α cells harboring the pBAD-T4L plasmid in the presence of 0.2 g/L L-arabinose. Lane M, standard marker protein; T, total cell extracts; S, soluble fraction of the total cell extracts; I, insoluble proteins from total cell lysates; P, purified T4L. Arrows indicate the expressed T4L protein.

Next, DH5α cells harboring the pBAD-T4L plasmid (Figure 3A) were grown in the presence of 0.02 g/L L-arabinose to induce T4L expression. The level and pattern of T4L expression were monitored over time (Figure 3B). Soluble T4L was expressed at all tested induction times ranging from 2 to 8 h. Induction for more than 4 h did not increase T4L expression (Figure 3B). Thus, T4L expression was induced for 4 h in all subsequent experiments. As expected, the control *E. coli* strain containing the empty pBAD24 plasmid did not express T4L (Figure 3B). To determine the optimal concentration for inducing T4L expression, the *E. coli* DH5α-pBAD-T4L strain was grown in 0.002–0.2 g/L L-arabinose. As the L-arabinose concentration increased, soluble T4L expression increased (Figure 3C). In the absence of L-arabinose, the cells showed no T4L expression, suggesting that T4L expression is tightly regulated by the P*_BAD_* promoter in *E. coli* DH5α cells. Finally, His6-tagged T4L was purified by immobilized metal affinity chromatography. Purified T4L showed a molecular mass of approximately 21 kDa, which is very similar to the theoretical molecular mass of His6-tagged T4L (Figure 3D). Purified T4L showed a specific activity of 662.18 ± 18.54 U/mg. 

### 2.3. T4L Expression in E. coli DH5α-GESS-CK Cells

**Figure 4:**
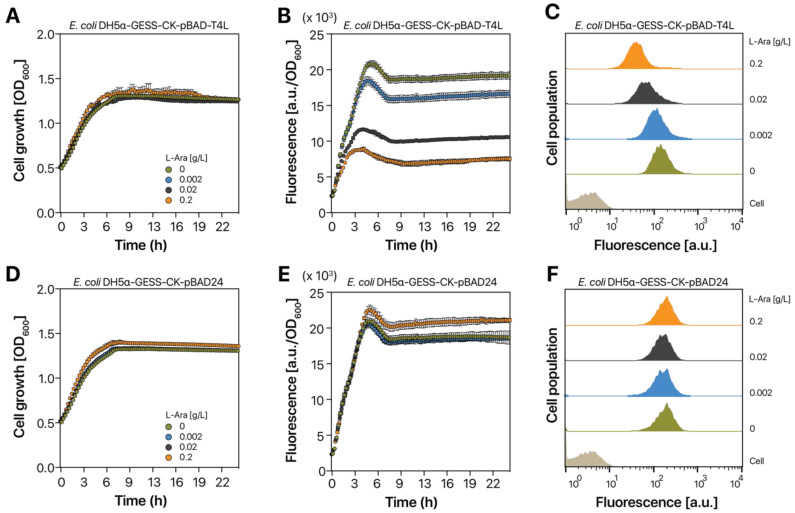
Effect of T4L expression on cell growth and fluorescence in response to induction with L-arabinose and *p*NP. Cell growth (OD_600_) of DH5α-GESS-CK-pBAD-T4L (**A**) and DH5α-GESS-CK-pBAD24 (**D**) in the presence of 0–0.2 g/L L-arabinose. Fluorescence output of DH5α-GESS-CK-pBAD-T4L (**B**) and DH5α-GESS-CK-pBAD24 (**E**) activated by 10 µM *p*NP at the population level. Fluorescence at the single-cell level was determined by flow cytometry using DH5α-GESS-CK-pBAD-T4L (**C**) and DH5α-GESS-CK-pBAD24 (**F**) induced by 10 µM *p*NP. Mean values of three biological replicates are shown. Error bars represent the standard deviation. “Cell” in panels C and F indicates the fluorescence of WT *E. coli* DH5α cells without the GESS system.

After confirming that T4L can be stably expressed in *E. coli* DH5α cells, we examined the effects of T4L expression on sfGFP fluorescence induced by the DmpR-CK variant recognizing *p*NP. To use DH5α-GESS-CK cells for HTS of T4L mutant libraries, T4L expression should not influence sfGFP fluorescence in DH5α-GESS-CK cells induced by *p*NP. We introduced the pBAD-T4L plasmid into DH5α-GESS-CK cells, which were induced with 10 µM *p*NP and 0–0.2 g/L L-arabinose to activate the GESS (i.e., sfGFP expression) and induce T4L expression, respectively. T4L expression did not inhibit growth of DH5α-GESS-CK cells induced by *p*NP (Figure 4A). Regardless of the L-arabinose concentration, the growth of DH5α-GESS-CK-pBAD-T4L cells was similar to that of the control strain, *E. coli* DH5α-GESS-CK-pBAD24 (Figure 4D). *E. coli* DH5α-GESS-CK-pBAD-T4L cells showed a significant difference in fluorescence (Figure 4B), whereas *E. coli* DH5α-GESS-CK-pBAD24 cells grown with L-arabinose showed no difference in fluorescence (Figure 4E). The fluorescence of *E. coli* DH5α-GESS-CK-pBAD-T4L cells decreased as the L-arabinose concentration increased. Because T4L expression is proportional to the L-arabinose concentration (Figure 3C), the reduced fluorescence of *E. coli* DH5α-GESS-CK-pBAD-T4L cells was caused by increased T4L expression. To examine the inhibitory effect of T4L expression on sfGFP fluorescence, we evaluated the fluorescence of *E. coli* DH5α-GESS-CK-pBAD-T4L cells by flow cytometry at the single-cell level (Figure 4C). Consistent with the results at the population level (Figure 4B), single-cell fluorescence was dose-dependently decreased after L-arabinose treatment, in contrast to the control strain (Figure 4F). 

### 2.4. HTS of a T4L Mutant Library

**Figure 5:**
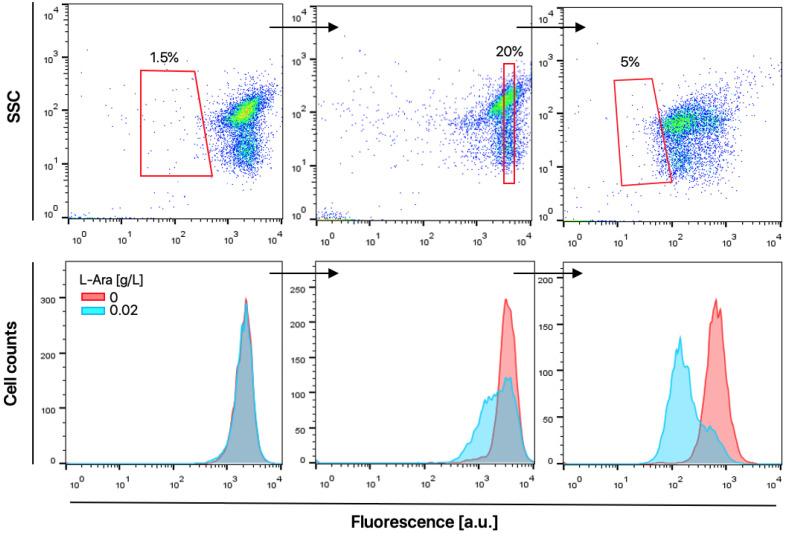
Screening of the T4L mutant library using a GESS-based genetic circuit and fluorescence-activated cell sorting (FACS) analysis. The red quadrangles in the upper panel indicate the gates of each FACS sorting round based on the double plot of side scatter (SSC) and fluorescence intensities. Gating included 1.5%, 20%, and 5% of all library populations, respectively. Representative histograms in the lower panel were obtained from the fluorescence profiles of the T4L mutant library in the absence and presence of 0.02 g/L L-arabinose.

Based on the observation that the sfGFP fluorescence of *E. coli* DH5α-GESS-CK-pBAD-T4L decreased as soluble T4L expression increased, we screened a T4L mutant library to identify T4L variants with improved lytic activity. First, we generated a random mutant T4L library using the error-prone PCR method. The resulting library was introduced into *E. coli* DH5α-GESS-CK cells and screened by negative sorting with a flow cytometer in the absence and presence of 0.02 g/L L-arabinose and 10 µM *p*NP (Figure 5). The flow cytometer sorting gate of three successive rounds was set at 1.5%, 20%, and 5% of all library populations, respectively (Figure 5, upper panel). Histograms obtained from each screening round showed that cell populations induced with 0.02 g/L L-arabinose were enriched at the position of lower fluorescence intensities compared to populations in the absence of L-arabinose (Figure 5, lower panel). To reduce false-positive clones in the library, a second sorting step was conducted in the absence of L-arabinose. After each round of sorting, plasmid DNA was isolated from the sorted cells and sequenced. 

**Figure 6:**
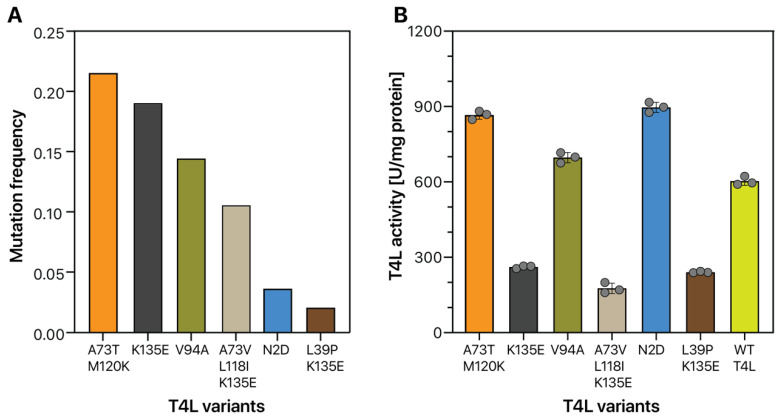
Isolated T4L variants identified using the GESS-CK genetic circuit. Among the 200 clones isolated by FACS analysis, 142 clones were successfully sequenced and categorized into six T4L mutant enzyme groups. The mutation frequency (**A**) was determined by dividing the number of clones with the corresponding mutation by the number of total sequenced clones. T4L activity (**B**) was determined using *Micrococcus lysodeikticus* cell walls labeled with fluorescein as a substrate at 37 °C for 30 min in the dark. Fluorescence was determined with a Victor X microplate reader equipped with a fluorescein filter.

Subsequently, we determined the DNA sequences of 200 clones isolated by FACS analysis. Among the 200 clones evaluated by Sanger sequencing, 142 clones contained full nucleotide sequences encoding T4L amino acids and showed 6 different T4L variants: A73T/M120K, K135E, V94A, A73V/L118I/K135E, N2D, and L39P/K135E (shown in order of high to low mutation frequency, Figure 6A). When we evaluated the activity of these six T4L variant enzymes, the lytic activity of N2D and A73T/M120K variants was improved by more than 1.48-fold (980.02 ± 21.85 U/ mg) and 1.43-fold (946.92 ± 17.88 U/mg), respectively, compared to native T4L (662.18 ± 18.54 U/mg). In a previous report, T4L mutants with improved activity showed amino acid mutations at the edge of helices and substitutions to flexible amino acids (glutamic acid and aspartic acid) [41]. Interestingly, the N2D and A73T/M120K variants screened from the random mutagenesis library of WT T4L using the GESS-CK genetic circuit contained similar substitutions at the N-terminal edge and within helices. The enzymatic activity of a mutant T4L containing an I3C mutation was unchanged [42]. However, the N2D mutant exhibited increased lytic activity compared to WT T4L. In accordance with the previous report [41], this result revealed that substitution of asparagine to a negatively charged flexible amino acid, aspartic acid, is another option for increasing the activity of bacteriophage T4L. Moreover, the A73T/M120K mutant showed increased flexibility at the mutation site, because threonine and lysine have higher B-factor values compared to alanine and methionine [41]. Based on these points, the A73T/M120K mutant obtained in this study had enhanced enzyme activity. To gain additional insight into how T4L double mutants (A73T/M120K) can increase enzymatic activity compared to WT T4L, additional structure-based analysis is required. However, our data presented indicate that genetic biosensor-based screening analysis, particularly using specific T4L characteristics, can be used to identify toxic protein variants with enhanced enzyme activity in microorganisms.

## 3. Conclusions

We designed and evaluated a GESS-based HTS method for bacteriophage T4L evolution, which can expand the applications of lysozyme from studies of protein structure–function relationships to biological degradation of secondary sludge. We used a designed substrate that releases a phenolic compound that is detected by the DmpR-based GESS. We also observed that increased soluble T4L expression decreased the fluorescence output of GESS cells. Based on this finding, we successfully screened a T4L mutant library and isolated T4L variant enzymes with improved activity. We will apply the system developed in this study to the evolution of other lysozyme types such as hen egg white lysozyme and human lysozyme, which have been widely used in foods, medicines, and environmental protection applications. Moreover, we will further examine the relationship between soluble T4L expression and cellular fluorescence of sfGFP in *E. coli* cells, which can lead to screening of additional T4L mutant libraries using a designed substrate and the GESS system.

## 4. Materials and Methods 

### 4.1. Plasmids and Bacterial Strains

The bacterial strains and plasmids used in this study are listed in Table 1. *E. coli* DH5α cells were used for plasmid construction and heterologous bacteriophage T4L expression. *E. coli* LMG194 cells were used to express and purify WT and mutant T4L. *E. coli* DH5α cells with chromosomally integrated GESS-CK were used for HTS of T4L variants with improved hydrolytic activity.

### 4.2. Protein Expression and Purification

Overnight cultures of *E. coli* strains containing the pBAD24 or pBAD-T4L plasmid were diluted with fresh lysogeny broth (LB) medium supplemented with 100 µg/mL ampicillin. The cells were further grown at 37 °C to an optical density value of 600 nm (OD_600_) of 0.4–0.5. At this time point, appropriate L-arabinose concentrations were added to induce lysozyme expression at 37 °C. Samples of the culture broth were collected intermittently. The culture broth was centrifuged at 16,000× *g* for 20 min at 4 °C to harvest the cells, which were resuspended in phosphate-buffered saline (PBS). Cell lysates were prepared by homogenizing the cell pellets using a sonicator (Thermo Fisher Scientific, Waltham, MA, USA) on ice. Soluble proteins were fractionated by recovering the supernatant after centrifuging the disrupted cells at 16,000× *g* at 4 °C for 20 min. Insoluble proteins were prepared by resuspending the remaining cell pellets in PBS. Approximately 10 µg of proteins were analyzed using 5–15% gradient polyacrylamide gels. To purify the expressed T4L, the homogenized cells were centrifuged at 16,000× *g* for 20 min at 4 °C, and the supernatant was filtered through a 0.45-µm filter. The filtered solution was applied to a Profinia protein purification system equipped with an immobilized metal affinity chromatography and desalting cartridge (Bio-Rad, Hercules, CA, USA). Protein concentrations were determined by a Bradford assay using bovine serum albumin as a standard.

### 4.3. Monitoring of Cell Growth and Fluorescence

*E. coli* DH5α-GESS-CK cells harboring the pBAD24 or pBAD-T4L plasmid were grown in 3 mL LB medium containing 100 µg/mL ampicillin in a 14-mL round-bottom tube at 37 °C while shaking at 200 rpm overnight. Cell cultures (50 µL) were transferred into 5 mL fresh LB medium containing 100 µg/mL ampicillin in a 50-mL baffled flask. The cultures were grown to an OD_600_ of 0.4–0.5 at 37 °C while shaking at 200 rpm. Next, 200 µL culture broth was inoculated into the wells of black-walled 96-well polystyrene microplates and incubated to monitor cell growth for 24 h at 37 °C with or without L-arabinose (10-fold dilutions from 0.2 to 0 g/L) in an Infinite 200 PRO microplate reader (Tecan, Männedorf, Switzerland). Fluorescence was simultaneously monitored to examine the effect of T4L expression on transcriptional activation of the DmpR-CK regulator in the *E. coli* DH5α GESS strain. Cell fluorescence was induced with L-arabinose supplemented with 10 µM *p*NP and measured at excitation and emission wavelengths of 488 and 515 nm, respectively. 

### 4.4. Flow Cytometry Analysis

To analyze fluorescence at the single-cell level, flow cytometry analysis was conducted using a FACS Calibur flow cytometer (BD Biosciences, Franklin Lakes, NJ, USA). *E. coli* DH5α-GESS-CK cells were induced with L-arabinose and 10 µM *p*NP for 8 h at 37 °C. Next, the cells were diluted by 1:100 with 1 mL PBS. Approximately 10,000 events were acquired at a low flow rate. To determine whether the P*_BAD_* promoter used in this study can be used for tunable expression of a heterologous protein in the *E. coli* DH5α strain, cells harboring pBAD-sfGFP were induced with 0–2 g/L L-arabinose at 37 °C for 8 h. Fluorescence was evaluated by flow cytometry; the data obtained were analyzed using FlowJo software (TreeStar, Ashland, OR, USA).

### 4.5. Random Mutagenesis Library Construction

Four to six mutations per 1000 bp of the T4L gene were induced via error-prone PCR using a Diversify PCR Random Mutagenesis Kit (Takara, Shiga, Japan). The pBAD-T4L plasmid was amplified using High-fidelity KOD-Plus-Neo polymerase (Toyobo) to prepare the backbone for the mutant library. The PCR products were purified by 1% agarose gel electrophoresis using a Wizard SV Gel and PCR Clean-Up System kit (Takara). PCR fragments were cloned into the backbone plasmid by Gibson assembly. The assembled mixture was cleaned up with a Wizard SV Gel and PCR Clean-Up system kit, and the entire product was transformed into *E. coli* DH5α electrocompetent cells. Once the transformed cells were grown in super optimal broth at 37 °C, an aliquot of cells was plated on LB solid medium supplemented with 100 µg/mL ampicillin to estimate the library size. The quality of the mutant library was verified by DNA sequencing. The remaining cultures were further grown at 37 °C on LB solid medium containing 100 µg/mL ampicillin and stored at −80 °C.

### 4.6. HTS of T4 Lysozyme Mutant Libraries by FACS

The error-prone T4L libraries were transformed into *E. coli* DH5α-GESS-CK electrocompetent cells, which were grown in super optimal broth medium for 1 h at 37 °C with shaking at 200 rpm. The cells were further grown on LB agar plates supplemented with 100 µg/mL ampicillin at 37 °C for 12 h. Colonies from the selection plates were scraped with LB medium, and this suspension was diluted by 1:100 in fresh LB medium containing 100 µg/mL ampicillin. The cells were grown at 37 °C with shaking at 200 rpm until the OD_600_ reached ~0.5. At that point, the cells were induced with 0.02 g/L L-arabinose supplemented with 10 µM *p*NP at 37 °C for 8 h. An aliquot of induced cells was washed with PBS, and FACS-based negative screening was conducted using a FACSAriaIII cell sorter (BD Biosciences). Negative screening was performed to isolate T4L variants showing reduced fluorescence compared to WT T4L. A recovered population was grown on LB agar plates supplemented with 100 µg/mL ampicillin. The remaining population was grown overnight in LB medium containing 100 µg/mL ampicillin for further screening rounds and storage at −80 °C. The T4L mutants were isolated and sequenced by Sanger sequencing to identify the enriched sequence variants.

### 4.7. Lysozyme Assay

Lysozyme activity was determined using an EnzChek Lysozyme Assay Kit (Thermo Fisher Scientific). Briefly, enzymatic reactions were carried out using *Micrococcus lysodeikticus* cell walls labeled with fluorescein as a substrate at 37 °C for 30 min in the dark. Fluorescence was determined with a Victor X microplate reader equipped with a fluorescein filter (PerkinElmer, Waltham, MA, USA). A standard curve was generated using hen egg white lysozyme and used to calculate T4L activity. Protein concentration was measured using the Bradford method with bovine serum albumin as a standard.

## Figures and Tables

**Table 1:** Bacterial strains and plasmids used in this study.

Strains or Plasmids	Description	Source
*E. coli* DH5α	F-, *Φ80lacZ·ΔM15·f(lacZYA-argF)U169 deoR recA1 endA1 hsdR17(rk,- mk+) phoA supE44 thi-1 gyrA96 relA1*	Thermo Fisher Scientific
*E. coli* LMG194	F- *ΔlacX74 gal E thi rpsL ΔphoA (Pvu II) Δara714 leu::*Tn10	Invitrogen
*E. coli* DH5α GESS-CK	DH5α derivatives with CK mutant *dmpR* GESS integrated into the chromosomal *bglA* locus of *E. coli* DH5α	[37]
pBAD/Myc-His/lacZ	Expression vector, P*_BAD_*:*lacZ*, pBR322 ori, Amp^R^	Invitrogen
pBAD24	Expression vector, P*_BAD_*, pBR322 ori, Amp^R^	Invitrogen
pBAD-sfGFP	*E. coli* codon-optimized superfolder GFP expressing plasmid, P_BAD_, pBR322 ori, Amp^R^	Addgene (Plasmid #85482)
pBAD-T4L	T4 lysozyme-expressing plasmid with an N-terminal His6 tag, Amp^R^	[43]
